# Effect of Reactant Addition Sequence on Maleic Anhydride Grafting onto Polylactic Acid During Peroxide-Initiated Melt Processing

**DOI:** 10.3390/ma19081488

**Published:** 2026-04-08

**Authors:** Seán Mulkerins, Guangming Yan, Noel Gately, Declan M. Devine, Keran Zhou, Caolan Jameson, Ciara Buckley, Amin Abbasi, Soheil Farshbaf Taghinezhad, Declan Mary Colbert

**Affiliations:** 1 PRISM Research Institute (Polymer, Recycling, Industrial, Sustainability and Manufacturing Engineering), Technological University of the Shannon, University Road, N37HD68 Athlone, Ireland; guangming.yan@tus.ie (G.Y.); declan.devine@tus.ie (D.M.D.); keranzhou96@gmail.com (K.Z.); ciara.buckley@tus.ie (C.B.); amin.abbasi@tus.ie (A.A.); soheil.farshbaf@tus.ie (S.F.T.); 2 Technology Transfer Office, Technological University of the Shannon, University Road, N37HD68 Athlone, Ireland; noel.gately@tus.ie

**Keywords:** polylactic acid, maleic anhydride, PLA-g-MAH, reactive melt processing, dicumyl peroxide, graft yield, acid–base titration

## Abstract

Maleic anhydride (MAH) grafting is widely employed to compatibilise polylactic acid (PLA) in fibre-reinforced composites; however, the influence of reactant addition sequence during melt processing varies widely across the literature, with no clear consensus on an optimal approach. In this study, the effect of reactant addition sequence on the graft yield of MAH onto PLA was investigated using dicumyl peroxide (DCP) as an initiator. Four loading protocols were examined in which the order of addition of PLA, DCP, and MAH was varied using approaches commonly reported in the literature, while all other processing conditions were held constant. A strong dependence of grafting yield on addition sequence was observed, with values ranging from 0.12% to 0.51%, corresponding to more than a four-fold variation under otherwise identical processing conditions. Simultaneous addition of PLA, DCP, and MAH produced the highest grafting yield, attributed to a more effective utilisation of peroxide-derived radicals. These results demonstrate that the reactant addition sequence is a critical processing variable governing MAH grafting efficiency and that simultaneous addition represents the most effective processing strategy under the conditions examined.

## 1. Introduction

Polylactic acid (PLA) is widely used as a bioabsorbable alternative to metals in biomedical applications, including orthopaedic fixation devices and vascular stents [1]. However, its comparatively lower mechanical properties hinder its broader adoption in these roles [2]. One widely recognised strategy to overcome this limitation is the formation of fibre-reinforced composites; however, the effectiveness of such composites can be constrained by sub-optimal interfacial adhesion [3].

To address the inherent incompatibility between the polymer matrix and the reinforcing fibre, a range of compatibilisation strategies has been employed. These include fibre surface treatments, such as alkali, silane, or plasma modification, which alter fibre surface chemistry and roughness to promote stronger interfacial interactions, as well as the use of coupling agents and reactive compatibilisers that introduce functional groups capable of interacting with both phases [4,5,6]. Among these, MAH grafting onto the PLA backbone (PLA-g-MAH) is commonly employed to enhance interfacial bonding and facilitate improved mechanical performance through the introduction of reactive functional groups [1]. Several studies have reported that MAH grafting improves interfacial adhesion and associated mechanical performance in PLA-based composites, with increases in tensile strength and modulus observed following grafting [7,8]. Therefore, given that MAH grafting is both widely adopted and effective, optimising the conditions under which it is performed is of particular importance.

MAH grafting onto PLA is commonly achieved via peroxide-initiated free-radical reactions during melt processing, where initiators such as DCP decompose to generate radicals that abstract hydrogen atoms from the PLA backbone, forming PLA macroradicals that can subsequently react with MAH [9]. The effectiveness of PLA-g-MAH is directly influenced by the efficiency of the grafting reaction, commonly quantified by the grafting yield, which determines the availability of anhydride functional groups for interfacial interactions [10]. To date, efforts to enhance grafting yield have focused predominantly on formulation parameters, particularly through variation in MAH and DCP concentrations and their relative proportion [7,10,11,12,13]. In contrast, the influence of processing variables has received comparatively less attention. Notably, the sequence of addition of PLA, initiator, and MAH during melt mixing varies widely across the literature, indicating that reactant sequencing remains an under-examined yet potentially influential processing parameter on graft yield.

One widely reported sequencing strategy involves first melting the PLA, adding the initiator (DCP) to generate radicals, and subsequently introducing MAH after 3–5 min [7,12,13]. An alternative approach is to reverse the order by introducing MAH before DCP [14]. The rationale typically offered for these staged approaches is that the first-added component is given time to disperse uniformly within the molten PLA before the second reactant triggers the grafting reaction, thereby promoting more homogeneous reaction sites throughout the matrix. In contrast to these staged approaches, additional studies have utilised a simultaneous approach in which DCP, MAH, and PLA are introduced together; however, the underlying rationale for this sequencing is not typically provided [11,15,16].

Reported grafting yields for the processing sequences discussed, although not always provided, are generally low and typically fall within the range of approximately 0.1–0.6 wt%, with variations within this range primarily attributed to differences in the MAH:DCP ratio used during processing [12,16,17,18,19]. As a result, although multiple sequencing strategies are reported in the literature, to our knowledge, no study has explicitly compared these strategies to determine how the order of addition influences graft yield independent of differences in MAH:DCP ratio. Consequently, the aim of this study is to investigate the effect of reagent addition sequencing on the graft yield of MAH onto PLA during melt processing using DCP as an initiator, to determine whether reactant addition order can meaningfully influence grafting efficiency.

## 2. Materials and Methods

### 2.1. Materials

PLA (Luminy^®^ LX175) was obtained from Total Corbion (Gorinchem, The Netherlands) in pellet form, with a density of 1.24 g/cm^3^ and a melt flow index of 8 g/10 min (210 °C/2.16 kg). The material is a semi-crystalline, hygroscopic polymer with a glass transition temperature (T_g_) of 60 °C and a melting temperature (T_m_) of 155 °C. Prior to processing, the pellets were dried in a Memmert oven at 85 °C for 6 hr in accordance with the supplier’s recommendations. The residual moisture content was reduced to below 0.01 wt%, as verified using an OHAUS MB90 moisture analyser (OHAUS Corporation, Parsippany, NJ, USA). MA and dicumyl DCP, both with a stated purity of 98%, were obtained from Sigma-Aldrich (Buchs, Switzerland).

### 2.2. Preparation of PLA-g-MAH

PLA-g-MAH was prepared using an internal mixer (Brabender MetaStation 4E, Brabender GmbH & Co. KG, Duisburg, Germany) equipped with roller blades (W50/W50EHT) operated at 190 °C and 55 rpm with a total mixing time of 10 min. The formulation was fixed at a PLA:DCP:MAH ratio of 100:0.6:4.5 phr for all batches. This composition was selected to fall within the mid-range of MAH and DCP concentrations commonly reported for PLA-g-MAH grafting [10,11,14,15,16,17,18,20,21]. Mixing was conducted in a 55 mL chamber using a fill factor of 80%, in accordance with the manufacturer’s recommendation, corresponding to a constant batch mass of approximately 54.6 g. For all trials, PLA was introduced into the mixing chamber via a continuous manual feed over approximately 1 min to allow for melting and compaction. DCP and MAH were introduced in accordance with the specified loading sequence (Table 1), either as discrete additions following PLA loading or incorporated during feeding under the simultaneous loading condition. The compounded material was discharged from the mixing chamber, air-cooled on a metal plate, and subsequently granulated into pieces of approximately 3 mm in size.

To isolate the effect of reactant addition sequence on grafting yield, four different loading protocols were investigated, as summarised in Table 1, while all other processing parameters were held constant. During each mixing run, torque–time data were recorded using the internal mixer to monitor melt behaviour and reaction progression, as discussed in Section 3.1.

### 2.3. Fourier Transform Infrared (FT-IR) Spectroscopy

FTIR spectroscopy equipped with an attenuated total reflectance (ATR) accessory was performed using a Thermo Scientific iZ10 Nicolet spectrophotometer (Braunschweig, Germany) over a spectral range of 4000–600 cm^−1^, with a resolution of 4 cm^−1^ and 16 scans per sample. FTIR analysis was conducted to confirm the presence of grafted anhydride functional groups in PLA-g-MAH. All PLA-g-MAH samples were purified prior to analysis using the same procedure described for the titration studies in Section 2.4. In addition to the grafted samples, both neat PLA and purified PLA were analysed to provide appropriate reference spectra, along with MAH for comparison.

### 2.4. Determination of Grafting Yield

The grafting yield of PLA-g-MAH was determined by acid–base titration based on measurement of the acid number, adapted from Jang et al. [17]. PLA-g-MAH samples (0.5 g) were dissolved in 50 mL of chloroform with the addition of 0.5 mL of 1 N hydrochloric acid and stirred at 500 rpm for 1 hr to ensure complete dissolution. The solution was then poured into 250 mL of methanol to precipitate the polymer and remove residual or unreacted MAH and DCP. The precipitated polymer was then collected by vacuum filtration and dried in a vacuum oven at 80 °C for 12 h.

For titration, the purified PLA-g-MAH sample was dissolved in 20 mL of a chloroform–methanol mixture (5:2, *v*/*v*) and stirred for 30 min. A 5 mL aliquot of this solution was transferred to a conical flask, and three drops of a 1 wt.% phenolphthalein solution in ethanol were added as an indicator. The solution was titrated with 0.02 N potassium hydroxide (KOH) in methanol until a persistent pink endpoint was observed. Acid number measurements were performed in triplicate for each batch, and the reported grafting yield was calculated using the mean acid number. The volume of KOH consumed (*V_KOH_*) and the corresponding grafting yield were calculated per Equations (1) and (2) [17,23]:

(1) $$Acid\;number\;(\mathrm{mg}\;KOH\;g^{-1})=\frac{\left(V_{KOH}\times N_{KOH}\times M_{KOH}\right)}{m}$$

(2) $$Grafting\;yield=\frac{\left(Acid\;number\times M_{MAH}\right)}{\left(2\times 561\right)}$$

where *V_KOH_* is the volume (mL) of KOH solution consumed, *N_KOH_* is the normality of the KOH solution, *M_KOH_* is the molar mass of KOH (g mol^−1^) (56.1 g mol^−1^), *M_MAH_* is the molar mass of maleic anhydride (98.06 g mol^−1^), and *m* is the mass of the polymer sample (g).

### 2.5. Thermal Properties

#### 2.5.1. Differential Scanning Calorimetry

Differential scanning calorimetry (DSC) was performed using a Pyris 6 instrument (PerkinElmer, Waltham, MA, USA). All measurements were conducted under a nitrogen purge at a flow rate of 30 mL min^−1^, using heating and cooling rates of 10 °C min^−1^. Heating–cooling–heating cycles were applied to all samples over a temperature range of 30–200 °C. The glass transition temperature (*T_g_*), cold-crystallisation temperature (*T_cc_*), and melting temperature (*T_m_*) were determined from the second heating scans to eliminate the influence of prior thermal history associated with melt processing. The degree of crystallinity (*X_c_*) was calculated from the enthalpy of melting (Δ*H_m_*) and the enthalpy of cold crystallisation (Δ*H_cc_*) using Equation (3):

(3) $$X_{c}\left(\%\right)=\frac{{∆H}_{m}-\;{∆H}_{cc}\;}{{∆H}_{m}^{0}}\;\times 100$$

where a ∆*H_m_*^0^ = 93 J·g^−1^ is used as the enthalpy of fusion for a 100% crystalline PLA [24].

#### 2.5.2. Thermogravimetric Analysis

Thermal degradation behaviour was evaluated using a high-resolution thermogravimetric analyser (TGA 5, TA Instruments, New Castle, DE, USA). Measurements were carried out under a nitrogen atmosphere, with samples heated from 30 to 700 °C at a constant rate of 10 °C min^−1^.

## 3. Results and Discussion

### 3.1. Torque Rheometry

Torque rheometry was used to monitor melt behaviour and changes in melt viscosity associated with reactant addition during each loading sequence. As described in Section 2.2, all samples were processed under identical conditions; therefore, differences in the torque–time profiles can be attributed primarily to variations in loading sequence.

#### 3.1.1. PLA Control Sample

**Figure 1:**
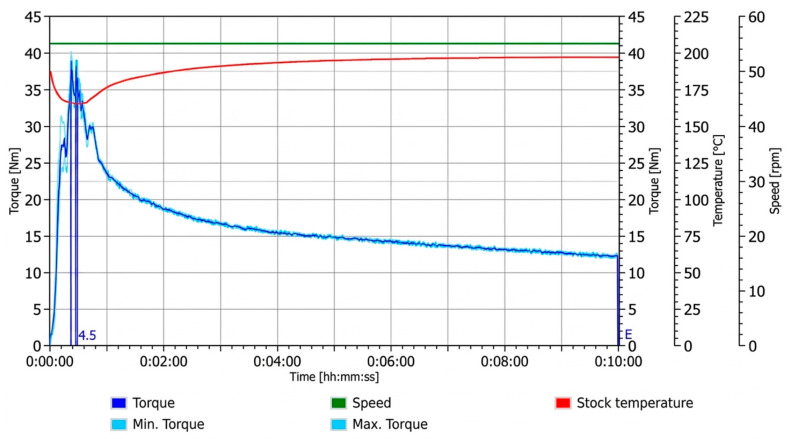
Torque–time response of the PLA control.

The processed PLA control (Figure 1) exhibited an expected initial sharp increase in torque during loading, corresponding to resistance from the solid material prior to complete melting. Upon reaching a fully molten state, a gradual and continuous decline in torque was observed over the remainder of the 10 min mixing period. This progressive reduction in torque reflects a decrease in melt resistance during prolonged processing and is consistent with behaviour reported for PLA under melt processing conditions [25]. Such trends have been attributed to thermally induced chain scission and associated reductions in molecular weight during extended residence times, which lead to decreased melt viscosity [26]. This response provides a baseline for comparison with torque profiles obtained following reactant addition and variations in loading sequence.

#### 3.1.2. DCP Addition Prior to MAH

**Figure 2:**
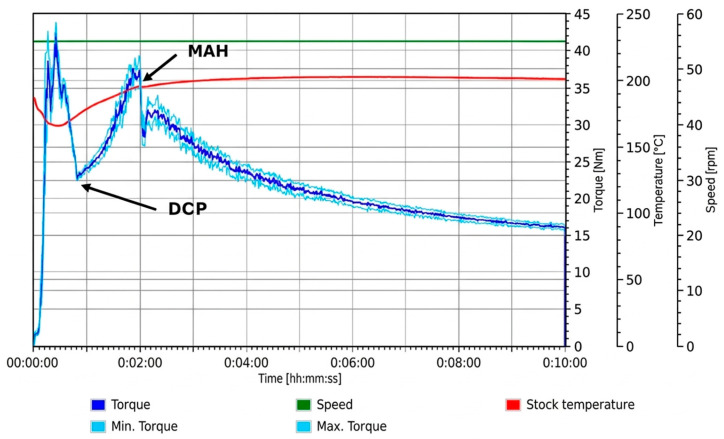
Torque–time response of Trial 1 during reactive melt processing, with DCP added directly after the PLA and MAH added at 2 min.

**Figure 3:**
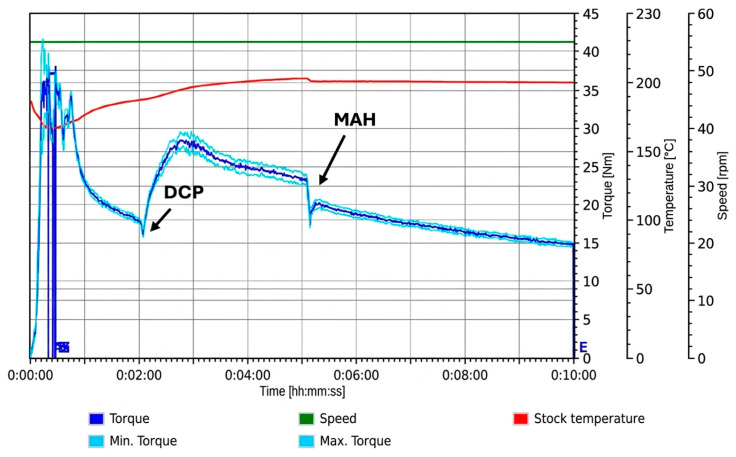
Torque–time response of Trial 2 during reactive melt processing, with DCP added at 2 min and MAH added at 5 min.

Following the initial torque rise associated with loading and melting of PLA, the addition of DCP in both trials—either immediately after PLA loading (Trial 1) (Figure 2) or after 2 min (Trial 2) (Figure 3)—produced a pronounced secondary increase in torque, indicating an increase in melt resistance. Comparable secondary torque increases have been reported during the reactive processing of PLA following DCP addition, where peroxide-induced radical formation results in a marked rise in torque after melting [22]. This behaviour is consistent with peroxide-initiated reaction within the PLA melt, which may include PLA–PLA reactions such as long-chain branching or crosslinking, leading to increased melt viscosity and torque [27]. Under these conditions, a fraction of peroxide-generated macroradicals is likely consumed through radical–radical interactions prior to the introduction of MAH, potentially reducing the population of radicals available for subsequent grafting reactions [19].

Upon subsequent introduction of MAH following DCP addition, a sharp reduction in torque was observed in both trials, followed by a moderate recovery and then a gradual decline over the remainder of the mixing cycle, with the latter trend consistent with the behaviour of the PLA control (Figure 1). The initial torque reduction is consistent with the presence of free, unreacted MAH, which has been reported to act as a plasticiser by increasing chain mobility and, thereby, reducing melt resistance [28]. The subsequent recovery in torque is attributed to the progressive consumption of MAH through grafting reactions with PLA macroradicals, diminishing its plasticising effect and coinciding with the formation of grafted side chains, which can restrict chain mobility and are consistent with the observed increase in torque [19].

#### 3.1.3. MAH Addition Prior to DCP

**Figure 4:**
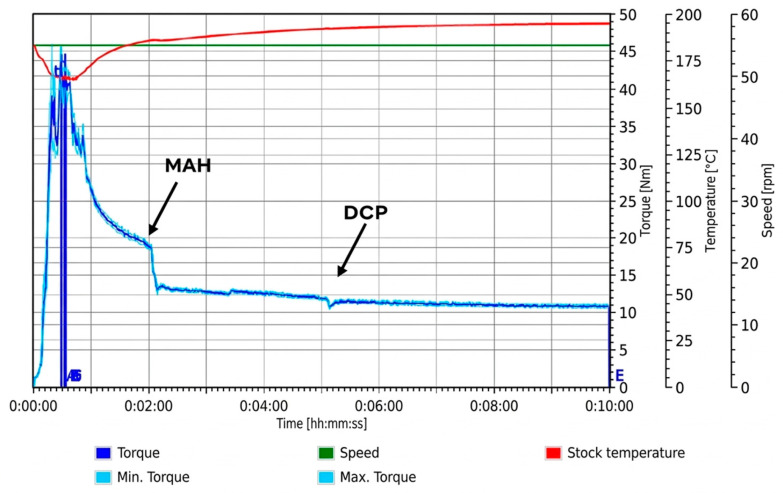
Torque–time response of Trial 3 during reactive melt processing, with MAH added at 2 min and DCP added at 5 min.

When MAH was introduced prior to DCP, a pronounced reduction in torque was observed, after which the torque remained relatively constant before gradually declining over the remainder of the mixing cycle (Figure 4). Although direct torque-based comparisons for MAH-first addition are limited in the literature, as already discussed, previous studies have reported that free, unreacted MAH can act as a plasticiser in PLA-based melts by increasing chain mobility, which provides a plausible explanation for the reduced melt viscosity observed during this loading sequence [28].

Subsequent addition of DCP at 5 min produced a minor reduction in torque, with no secondary torque increase observed, in contrast to the DCP-first loading routes. This behaviour suggests that when MAH is present prior to peroxide addition, peroxide-initiated radical chemistry is more favourably directed toward grafting reactions rather than PLA–PLA reactions associated with a pronounced increase in melt viscosity via long-chain formation, branching, or crosslinking.

#### 3.1.4. Simultaneous Mixing

**Figure 5:**
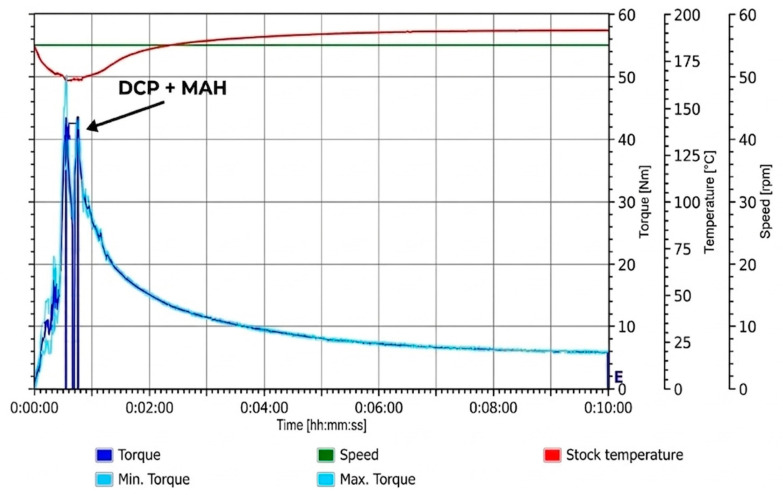
Torque–time response of Trial 4 during reactive melt processing, with PLA, DCP, and MAH simultaneously.

Simultaneous addition of PLA, DCP, and MAH produced a torque–time profile closely resembling that of the PLA control, with no pronounced secondary torque features observed (Figure 5). To our knowledge, torque–time responses for the simultaneous addition of PLA, DCP, and MAH have not been previously reported. However, the absence of distinct torque features suggests that competing peroxide-induced PLA–PLA reactions, as well as plasticising effects from free, unreacted MAH, are minimised under these conditions. This indicates a more efficient utilisation of peroxide-derived radicals and MAH, compared with the staged loading sequences. As discussed later in Section 3.2, this processing route resulted in a notably higher grafting yield compared to all staged addition methods. Taken together, these observations are consistent with a more effective utilisation of peroxide-derived PLA macroradicals for grafting when DCP and MAH are present simultaneously, rather than their consumption via competing PLA–PLA reactions.

However, as shown in Figure 5 and summarised in Table 2, the simultaneous loading condition (Trial 4) exhibited the lowest final torque of all formulations.

**Figure 6:**
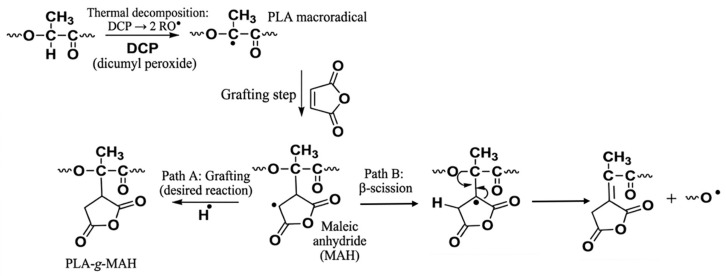
Reaction mechanism for DCP-initiated grafting of maleic anhydride (MAH) onto PLA during melt processing, illustrating the formation of PLA macroradicals, subsequent MAH grafting (Path A), and competing β-scission reactions (Path B), adapted from [14].

A similar reduction in torque during reactive processing of PLA-g-MAH has been reported in the literature, where lower torque in the grafted polymer relative to neat PLA was attributed to molecular weight reduction arising from β-chain scission during peroxide-initiated grafting [14]. The study noted that under DCP initiation, radical formation enables MAH grafting onto the PLA backbone, while also promoting β-chain scission (as can be seen in Figure 6), which can reduce molecular weight and, consequently, melt viscosity. In this context, the lower final torque observed for Trial 4 is consistent with a greater extent of β-chain scission relative to the other trials, despite the higher graft yield obtained under this condition.

### 3.2. Acid-Base Titration

Grafting yields of PLA-g-MAH were determined by acid–base titration and are summarised in Table 3. Acid numbers were measured in triplicate, and graft yield was calculated for each replicate. A strong dependence of grafting yield on component addition sequence was observed, with values ranging from 0.12% to 0.51% across the four processing routes, representing more than a four-fold variation under otherwise identical processing conditions. A one-way ANOVA indicated a statistically significant effect of processing route on graft yield (F = 10.68, *p* = 0.004). Pairwise comparisons using Fisher’s least significant difference (LSD) test indicated that Trial 4 exhibited significantly higher graft yield than all other conditions. In addition, Trial 1 showed significantly lower graft yield than both Trial 2 and Trial 3, while no statistically significant difference was observed between Trials 2 and 3.

Simultaneous addition of PLA, DCP, and MAH produced the highest grafting yield (0.51%), almost twice that obtained for the staged addition routes in Trial 2 (0.29%) and Trial 3 (0.28%), and more than four times that observed for Trial 1 (0.12%). This indicates that concurrent availability of the initiator and MAH during melt mixing is the most favourable condition for effective graft formation.

In contrast, DCP-first staged addition routes exhibited markedly different graft yields, demonstrating that the timing of DCP addition has a pronounced effect on overall graft yield. In particular, the immediate addition of DCP following PLA loading in Trial 1 resulted in the lowest grafting yield (0.12%), substantially lower than all other processing routes. This identifies early peroxide exposure, prior to full melting of the PLA, as a distinctly unfavourable condition for effective graft formation.

The MAH-first route (Trial 3) produced a grafting yield of 0.28%, comparable to that obtained for Trial 2. In this processing sequence, DCP was introduced after MAH addition, thereby reducing the opportunity for peroxide-initiated PLA–PLA reactions to occur prior to MAH availability. Under such conditions, a greater fraction of peroxide-derived radicals could be expected to participate in grafting reactions between PLA and MAH rather than being consumed through PLA–PLA reactions. However, the grafting yield obtained for Trial 3 did not exceed that of Trial 2 and remained substantially lower than that achieved via simultaneous addition.

A plausible explanation for this behaviour is the reduced duration of melt mixing following initiator addition. In the simultaneous loading route, all components were present and allowed to react over the full 10 min mixing period, whereas in Trial 3, mixing continued for only 5 min after DCP introduction. While direct studies on the influence of mixing time on PLA-g-MAH graft yield are limited, related peroxide-initiated grafting studies on PLA have demonstrated the existence of an optimal processing time, with variations in mechanical performance attributed to changes in graft yield as a function of mixing duration [29]. This suggests that the mixing duration employed in Trial 3 may not be optimal and that higher graft yields could be achievable under longer post-initiator mixing times.

### 3.3. Fourier Transform Infrared Spectroscopy

**Figure 7:**
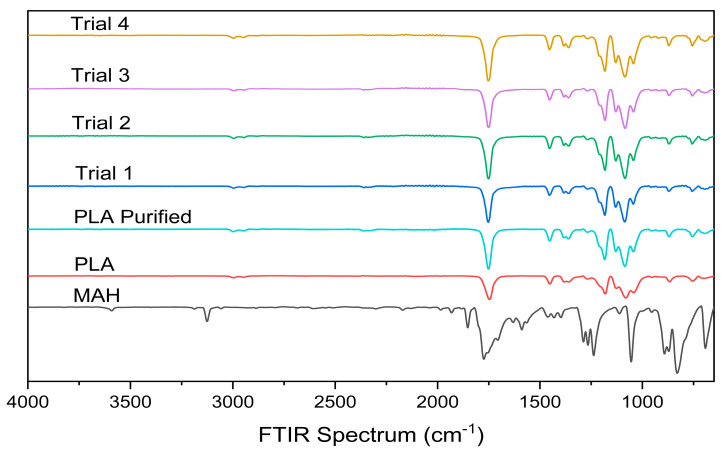
FTIR spectra of neat PLA, purified PLA MAH, and reactively processed samples from Trials 1–4.

Fourier Transform Infrared Spectroscopy (FTIR) analysis was performed to confirm the presence of MAH grafting onto the PLA backbone. The resulting FTIR spectra for neat PLA, purified PLA, MAH, and the reactively processed samples from Trials 1–4 are shown in Figure 7.

**Figure 8:**
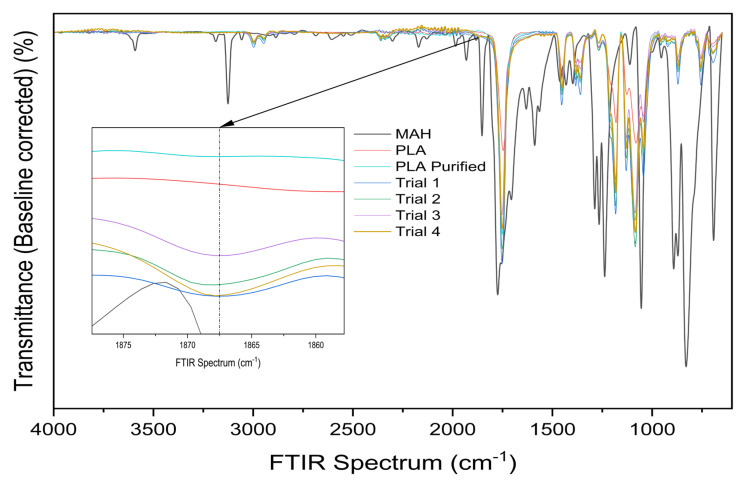
FTIR spectra of neat PLA, purified PLA, and reactively processed samples from Trials 1–4. The inset highlights the carbonyl region (~1865–1870 cm^−1^) [9,17].

All grafted samples exhibited the emergence of a band centred between 1865 cm^−1^ and 1870 cm^−1^ that was absent in the PLA control and purified PLA sample (Figure 8). The appearance of this band is consistent with reports in the literature for PLA-g-MAH systems, where it has been attributed to the asymmetric C=O stretching of the anhydride group [9,30,31,32]. The absence of this feature in both the neat and purified PLA samples, combined with its consistent appearance across all grafted formulations, supports the conclusion that MAH grafting occurred, in agreement with the trends observed in the titration-derived grafting yields.

### 3.4. Thermal Analysis

#### 3.4.1. Differential Scanning Calorimetry

**Figure 9:**
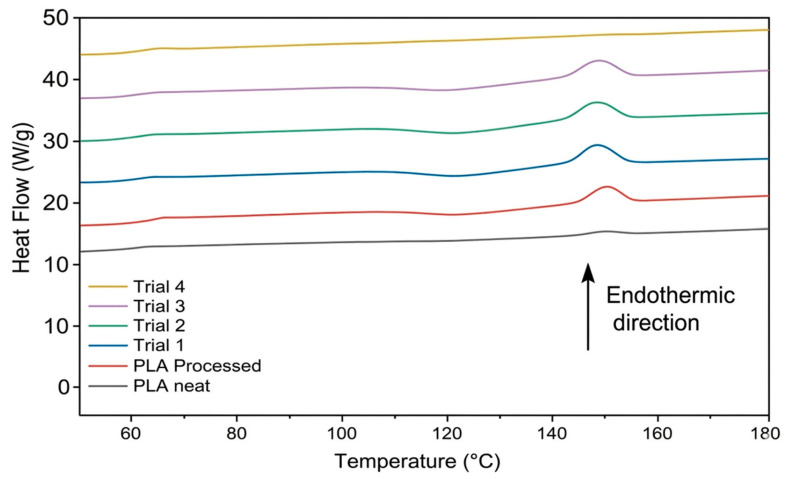
DSC heating thermograms of neat PLA, processed PLA, and reactively processed samples from Trials 1–4, shown with a vertical offset for clarity.

DSC was employed to characterise the thermal behaviour of the prepared samples. The thermograms presented correspond to the second heating scan, following an initial heating–cooling cycle used to erase prior thermal history from processing. Figure 9 shows the DSC heating thermograms of neat PLA, processed PLA, and reactively processed samples. Heat flow was normalised to sample mass and is presented with endothermic transitions plotted upward. The resulting thermal parameters are summarised in Table 4. Given the small magnitude of the observed differences, the results are interpreted as qualitative numerical trends rather than statistically distinct effects.

##### Processed PLA

For the processed PLA control, melt processing resulted in only moderate changes in thermal transition behaviour. The T_g_ increased slightly from 58.65 °C in virgin PLA to 61.56 °C following processing, while the T_m_ increased marginally from 147.74 °C to 148.69 °C. The degree of crystallinity after melting remained broadly similar, increasing from 4.44% to 5.22%. In addition, a cold crystallisation peak was observed following melt processing, which was not present in the neat PLA sample. The emergence of a cold crystallisation peak following melt processing has been reported previously by Pantani et al., who attributed this behaviour to an increased crystallisation rate arising from the presence of a limited fraction of lower-molecular-weight chains generated during processing [33]. These shorter chains act as nucleation sites, promoting crystallisation upon reheating. Consequently, the appearance of cold crystallisation in the present study is consistent with the formation of a limited lower-molecular-weight fraction attributed to processing-induced degradation.

##### DCP Addition Before MAH

The incorporation of DCP and MAH in both Trial 1 and Trial 2 resulted in a progressive reduction in thermal transition temperatures and crystallinity relative to the processed PLA control. Trial 1 exhibited a reduction in T_g_ from 61.56 °C to 59.70 °C and a decrease in T_m_ to 146.85 °C from 148.69 °C, accompanied by a reduction in crystallinity from 5.22% to 4.27%. These changes suggest that even low levels of grafting (0.18%) may introduce a measurable disruption to chain packing and crystalline organisation. This behaviour is consistent with reports in the literature showing that even slight increases in MAH grafting are associated with similarly slight reductions in thermal transition temperatures and crystallinity due to disruption of chain regularity following the introduction of MAH side groups [19,34].

Trial 2 showed a further reduction in T_g_ to 58.72 °C and T_m_ to 146.36 °C, together with a shift in T_cc_ from 121.92 °C to 120.60 °C and a more pronounced decrease in crystallinity to 3.44%. These changes are consistent with the higher graft yield achieved in Trial 2 (0.29%), suggesting a stronger reduction in crystallisation with increasing extent of grafting.

##### MAH Addition Before DCP

Relative to processed PLA, Trial 3 showed a reduction in T_g_ and crystallinity relative to the processed PLA control, consistent with graft-induced disruption of chain regularity arising from the introduction of MAH side groups. T_g_ decreased to 59.59 °C (vs. 61.56 °C) and crystallinity to 3.48% (vs. 5.22%), while T_m_ remained comparable at 147.18 °C. When MAH was introduced prior to DCP initiation, the resulting thermal behaviour was broadly comparable to that of Trial 2, in line with their similar graft yields of 0.28% and 0.29%, respectively. A modest reduction in T_cc_ from 120.60 °C to 118.25 °C was observed, suggesting a minor alteration in crystallisation rate; however, the final crystallinity and thermal transition temperatures remained essentially unchanged.

##### Simultaneous Loading

Trial 4, produced via simultaneous addition of PLA, DCP, and MAH, exhibited thermal behaviour that was markedly different from all other samples. In contrast to Trials 1–3, no cold crystallisation was observed during reheating, and the melting enthalpy was substantially reduced to 1.06 J·g^−1^, resulting in a low degree of crystallinity (1.15%). This represents a substantial reduction relative to the crystallinity values of approximately 3–5% observed for all other processed samples. To our knowledge, the crystallisation behaviour observed by DSC for PLA-g-MAH prepared via simultaneous addition of PLA, DCP, and MAH during melt processing has not been reported in the literature. Nonetheless, the results indicate that crystallisation is strongly inhibited under the applied thermal conditions, with the polymer chains unable to reorganise into ordered crystalline domains within the DSC time–temperature window.

Trial 4 also achieved the highest graft yield (0.51%), indicating that this behaviour is associated with a greater extent of graft-induced modification relative to the staged addition routes. However, T_g_ (60.12 °C) and T_m_ (148.34 °C) remained broadly comparable to those of the other processed samples, suggesting that the observed response is consistent with inhibited crystallisation rather than a general alteration of bulk thermal transition behaviour.

#### 3.4.2. Thermogravimetric Analysis

**Figure 10:**
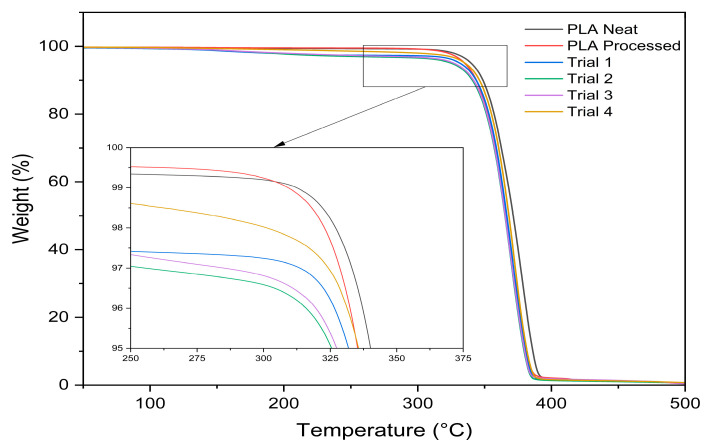
TGA weight–temperature curves of neat PLA and reactively processed samples from Trials 1–4.

The thermal degradation behaviour of samples was evaluated by TGA, as shown in Figure 10.

All processed trials showed a slight reduction in thermal stability relative to neat PLA across all degradation metrics (T_10_, T_25_, T_50_, and T_max_), as shown in Table 5. This behaviour is consistent with previous reports on peroxide-initiated reactive processing of PLA and is commonly attributed to peroxide-induced chain scission, which leads to a reduction in molecular weight and an earlier onset of thermal degradation [14].

For Trials 1–3, mass loss was observed to initiate at approximately 150 °C, which was not evident in Trial 4. A similar two-stage degradation behaviour has been reported for PLA–MAH systems, where an initial mass reduction occurs between the degradation temperatures of neat MAH (~120 °C) and neat PLA (~350 °C). This early-stage mass loss has been attributed to the presence of unreacted MAH, which volatilises or decomposes at lower temperatures than the PLA backbone [35]. In the present study, the more pronounced early mass loss observed for Trials 1–3 is therefore likely associated with a higher concentration of unreacted MAH arising from lower grafting efficiency. In contrast, the absence of this feature in Trial 4 is consistent with its higher graft yield and suggests a reduced fraction of unreacted MAH.

Trial 4 also exhibited slightly higher thermal stability across all degradation metrics compared with the staged addition sequences. This behaviour correlates with the higher graft yield achieved under simultaneous loading. While all samples show evidence of peroxide-induced chain scission, the results suggest that the simultaneous addition route may reduce its overall impact on thermal stability.

### 3.5. Visual Observations

**Figure 11:**
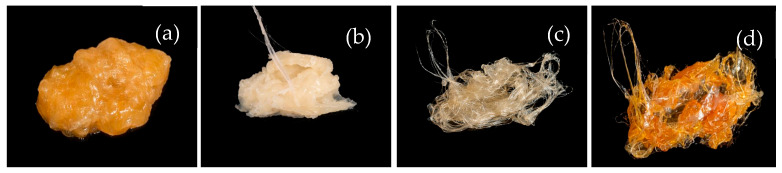
Photographs of the as-mixed material obtained following Brabender internal mixing for each processing route, prior to granulation: (**a**) Trial 1, (**b**) Trial 2, (**c**) Trial 3, and (**d**) Trial 4.

Clear qualitative differences in the appearance of the as-mixed materials were observed between the four processing routes, as shown in Figure 11.

With respect to colour variation, a clear correlation was observed between the extent of discolouration and the timing of DCP addition. Samples in which DCP was introduced at the onset of mixing (Trials 1 and 4) exhibited the most pronounced yellow–orange discolouration, whereas delayed addition produced progressively lighter samples. For example, Trial 2, in which DCP was added after 2 min, appeared pale yellow, while Trial 3 (DCP added after 5 min) exhibited only slight yellow discolouration.

Yellow–orange discolouration during peroxide-initiated melt grafting of PLA has been widely reported and is commonly attributed to thermal and radical-induced degradation during melt processing [4,36]. In this regard, Nyambo et al. observed that radical-driven degradation correlated with increased yellowing and was accompanied by reductions in molecular weight and tensile properties when maleated PLA was used to form a fibre composite [5]. As a result, the more pronounced yellowing observed in Trials 1 and 4 may be indicative of a greater extent of degradation during processing. This suggests that, although Trial 4 demonstrated the highest graft yield, the loading strategy may also promote radical-driven side reactions associated with degradation, potentially influencing its subsequent effectiveness as a composite material.

Regarding physical behaviour, qualitative variations in the physical response of the materials were also observed (Figure 10). Trials 1 and 2 (DCP first) exhibited a softer, elastomeric behaviour, whereas Trials 3 and 4 were noticeably more brittle. To our knowledge, the effects of loading sequence on the physical properties of PLA-g-MAH have not been investigated. However, the combined physical and colour observations indicate that the reagent addition sequence is a critical processing variable influencing not only graft yield but also the broader macroscopic behaviour of the resulting material.

## 4. Conclusions

The aim of this study was to investigate the influence of reactant addition sequence on the graft yield of MAH onto PLA using DCP as an initiator. A strong dependence of graft yield on addition sequence was demonstrated, with values ranging from 0.12% to 0.51% under otherwise identical processing conditions. Simultaneous addition of PLA, DCP, and MAH produced the highest graft yield, approximately two-fold higher than that achieved via staged addition routes and more than four times higher than that obtained when DCP was added immediately after PLA melting. These results demonstrate that the reactant addition sequence is a critical processing variable governing MAH grafting efficiency during peroxide-initiated melt processing of PLA.

While these results establish the importance of sequencing, several limitations were encountered that should be addressed in future work. Specifically, only a single DCP:MAH ratio was examined. Although this enabled isolation of the effect of reactant addition sequence, the conclusions regarding sequencing effects are, therefore, conditional on the selected formulation window. Future studies should examine a broader range of DCP:MAH ratios to determine the general applicability of the observed trends.

Pronounced differences in thermal behaviour were also observed for the simultaneous loading route, although the underlying cause of these differences was not explicitly resolved. In particular, this loading sequence exhibited strong inhibition of crystallisation, consistent with its higher graft yield. However, T_g_ and T_m_ remained broadly unchanged, deviating from the more typical trend of decreasing thermal transition temperatures with increasing graft content. This indicates that graft yield alone does not fully account for the observed thermal response, and further investigation is required to better understand the governing mechanisms.

Furthermore, the observed variations in colour intensity and qualitative mechanical behaviour indicate that the reagent addition sequence may influence radical-induced structural changes beyond graft formation. Additionally, differences in torque profiles and stabilised torque values between formulations suggest variations in melt viscosity, which may be associated with changes in molecular weight arising from competing grafting and degradation reactions. Therefore, direct assessment of molecular weight and quantitative mechanical performance would be valuable to clarify the relationship between graft efficiency, degradation, and mechanical performance. In particular, the absence of direct molecular weight characterisation (e.g., GPC/SEC or viscosity-based analysis) represents a limitation of the present study. Future work should incorporate such techniques to better quantify the influence of processing sequence on molecular weight during reactive processing. In summary, this study demonstrates that the reactant addition sequence exerts a clear influence on MAH grafting efficiency. Given the diversity of sequencing strategies reported in the literature, these findings underscore the importance of explicitly defining and controlling reagent addition order during peroxide-initiated melt processing.

## Figures and Tables

**Table 1:** Summary of reactant loading sequences and mixing protocols employed for the preparation of PLA-g-MAH. Arrows (→) denote sequential addition of components, while the addition symbol (+) indicates components introduced simultaneously.

Trial	Loading Sequence	Mixing Protocol	Refs.
Control	PLA only	PLA mixed for 10 min	n/a
1	PLA + DCP → MAH	PLA loaded; DCP added immediately and mixed for 2 min; MAH added and mixed for the remaining 8 min	[17]
2	PLA → DCP → MAH	PLA mixed for 2 min; DCP added and mixed for 3 min; MAH added and mixed for the final 5 min	[12,13,22]
3	PLA → MAH → DCP	PLA mixed for 2 min; MAH added and mixed for 3 min; DCP added and mixed for the final 5 min	[14]
4	PLA + DCP + MAH	PLA, DCP, and MAH pre-mixed by tumble mixing for 3 min prior to loading and introduced simultaneously, followed by mixing for the remainder of the 10 min cycle	[11,15,16]

**Table 2:** Stabilised torque values for PLA and PLA-g-MAH samples prepared using different addition sequences.

Sample	Stabilised Torque (Nm)
PLA	12.34
Trial 1	16.14
Trial 2	14.96
Trial 3	10.78
Trial 4	5.88

**Table 3:** Grafting yield determined for each processing trial following reactive melt processing.

Batch	Trial 1	Trial 2	Trial 3	Trial 4
Acid number (mg KOH g^−1^)	4.36 ± 0.53	6.29 ± 0.54	6.20 ± 1.07	8.85 ± 1.44
Graft yield (%)	0.12 ± 0.05	0.29 ± 0.05	0.28 ± 0.09	0.51 ± 0.13

**Table 4:** DSC-derived thermal properties of virgin PLA, processed PLA, and reactively processed samples from Trials 1–4.

Material	T_g_ (°C)	ΔH_cc_ (J·g^−1^)	T_cc_ (°C)	ΔH_m_ (J·g^−1^)	T_m_ (°C)	X_c_ (%)
PLA Neat	58.65	0	0	4.13	147.74	4.44
PLA Processed	61.56	14.38	121.75	19.23	148.69	5.22
Trial 1	59.70	15.91	121.92	19.79	146.85	4.27
Trial 2	58.72	15.55	120.60	18.75	146.36	3.44
Trial 3	59.59	15.57	118.25	18.81	147.18	3.48
Trial 4	60.12	0	0	1.06	148.34	1.15

**Table 5:** Temperatures corresponding to 10%, 25%, and 50% mass loss (T_10_, T_25_, and T_50_), maximum degradation temperature (T_max_), and residual mass at 500 °C (residue %) for neat PLA and reactively processed samples from Trials 1–4.

Sample	T_10_	T_25_	T_50_	T_max_	Residue %
PLA Neat	348.73	360.06	371.40	377.66	0.08
PLA Processed	343.79	355.29	365.96	368.18	0.01
Trial 1	343.87	356.37	366.71	368.54	0.08
Trial 2	340.81	354.48	365.15	368.58	0.09
Trial 3	341.67	354.83	365.33	368.87	0.05
Trial 4	346.17	358.00	368.17	372.61	0.09

## Data Availability

The original contributions presented in this study are included in the article. Further inquiries can be directed to the corresponding authors.
